# Supplementary material for: Measuring childhood maltreatment: Psychometric properties of the Norwegian version of the Maltreatment and Abuse Chronology of Exposure (MACE) scale
Source: PLoS One. 2020 Feb 27;15(2):e0229661. doi: 10.1371/journal.pone.0229661 (PMC7046287; doi:10.1371/journal.pone.0229661)
Supplement: S1 File — (DOCX) [file pone.0229661.s001.docx]

**S1  *Figures from Rasch analyses for 10 MACE subscales***


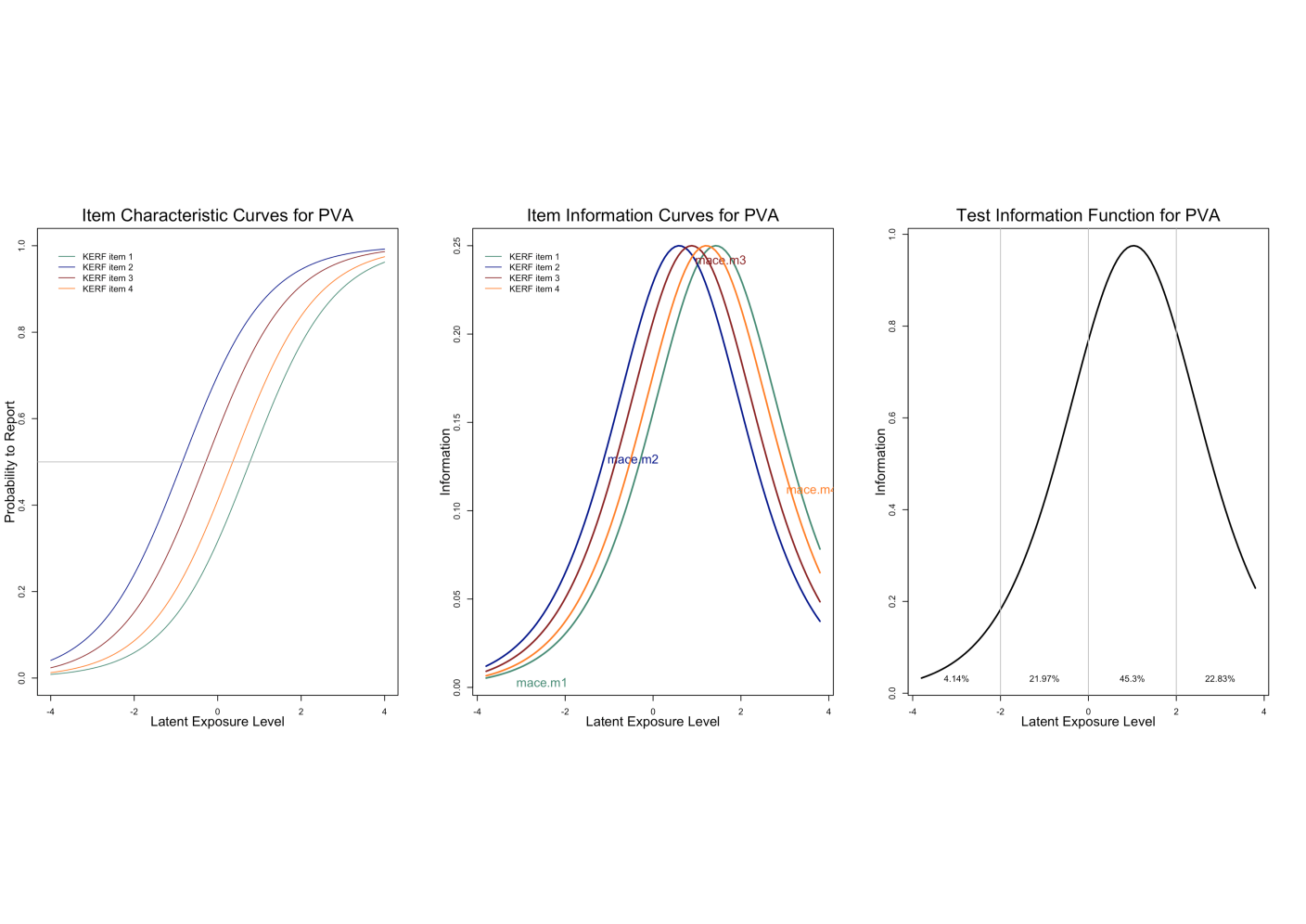
Figure A. Parental Verbal Abuse (PVA)


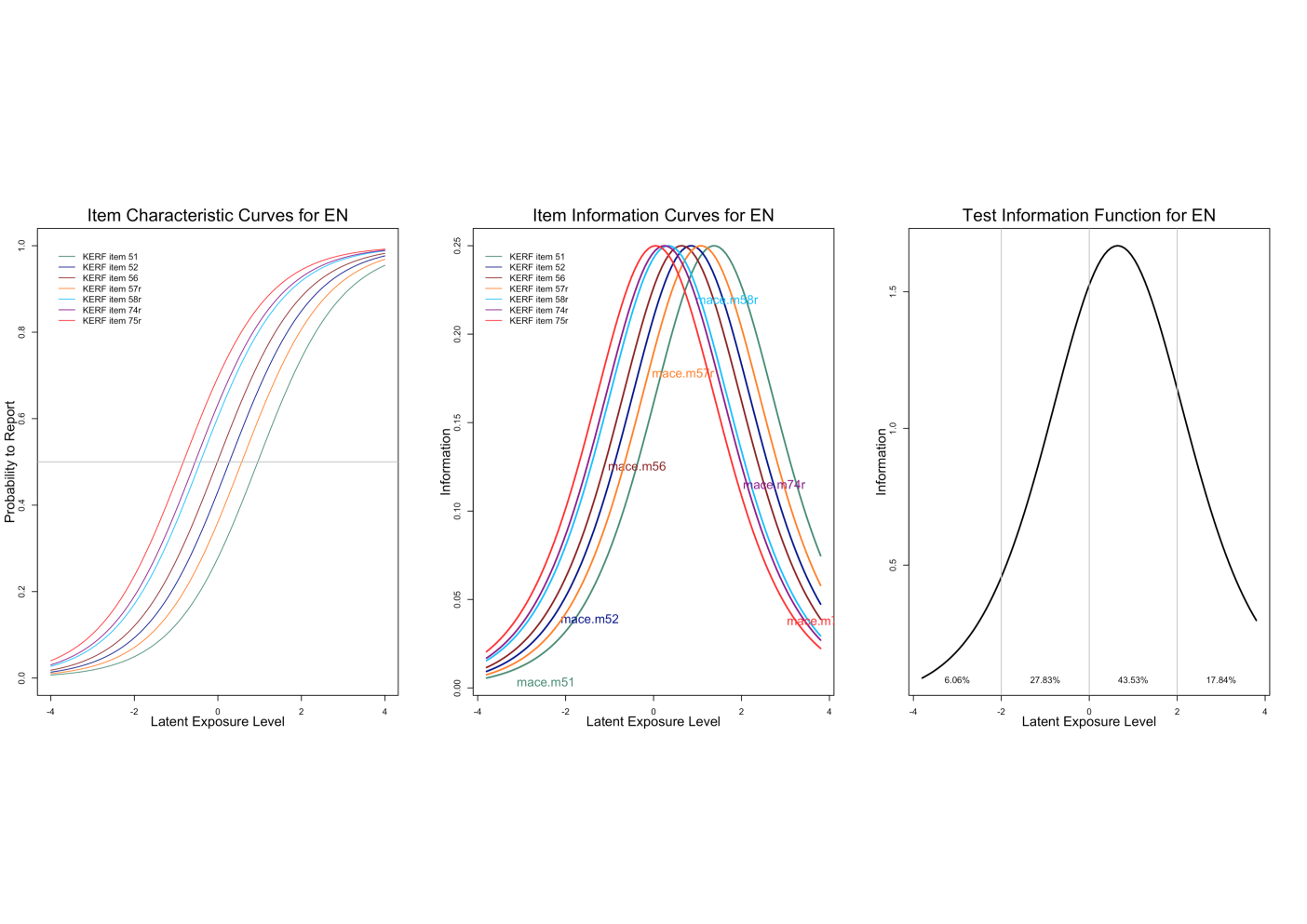
Figure B. Emotional neglect (EN)

Figure C. Physical neglect (PN)


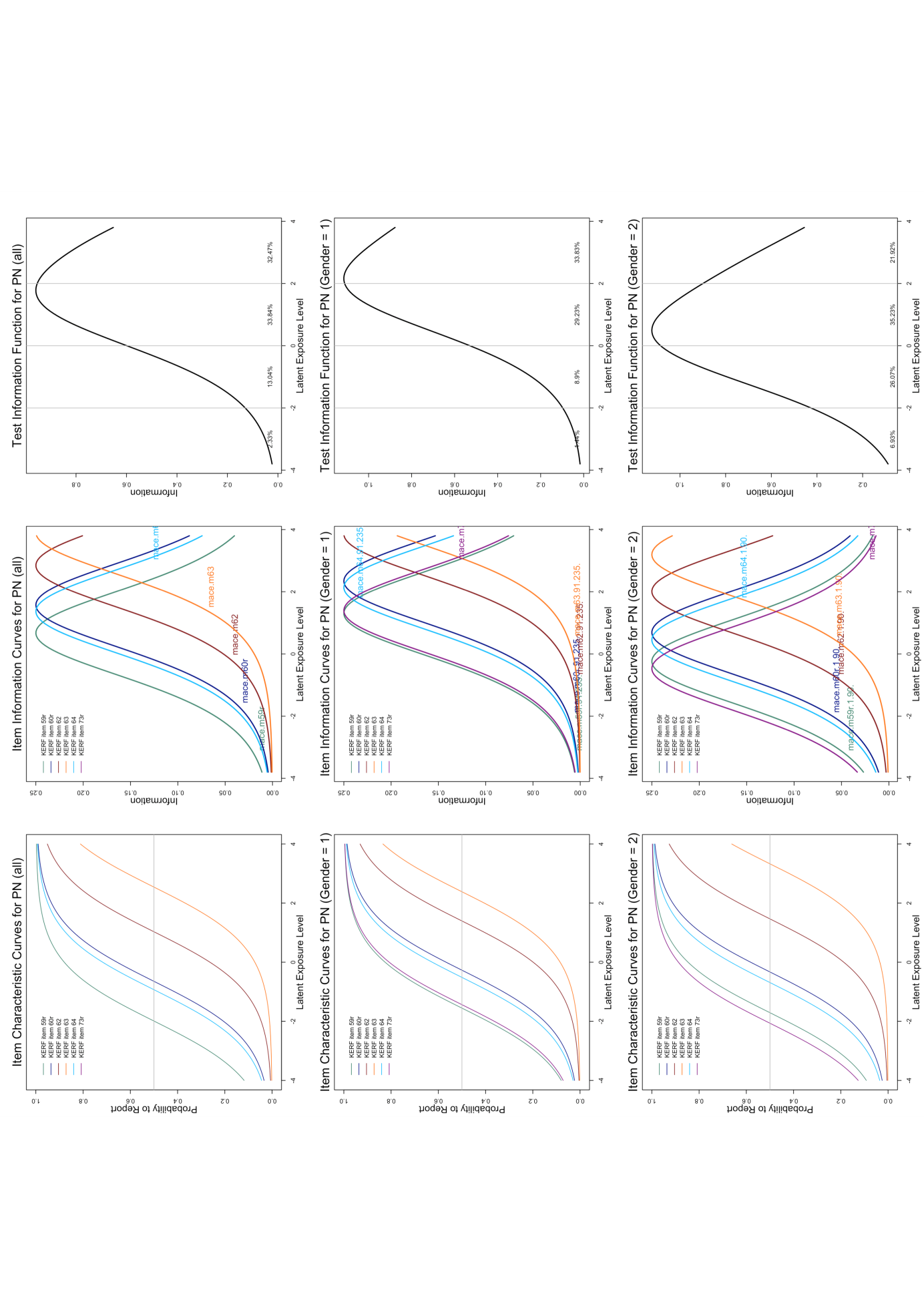


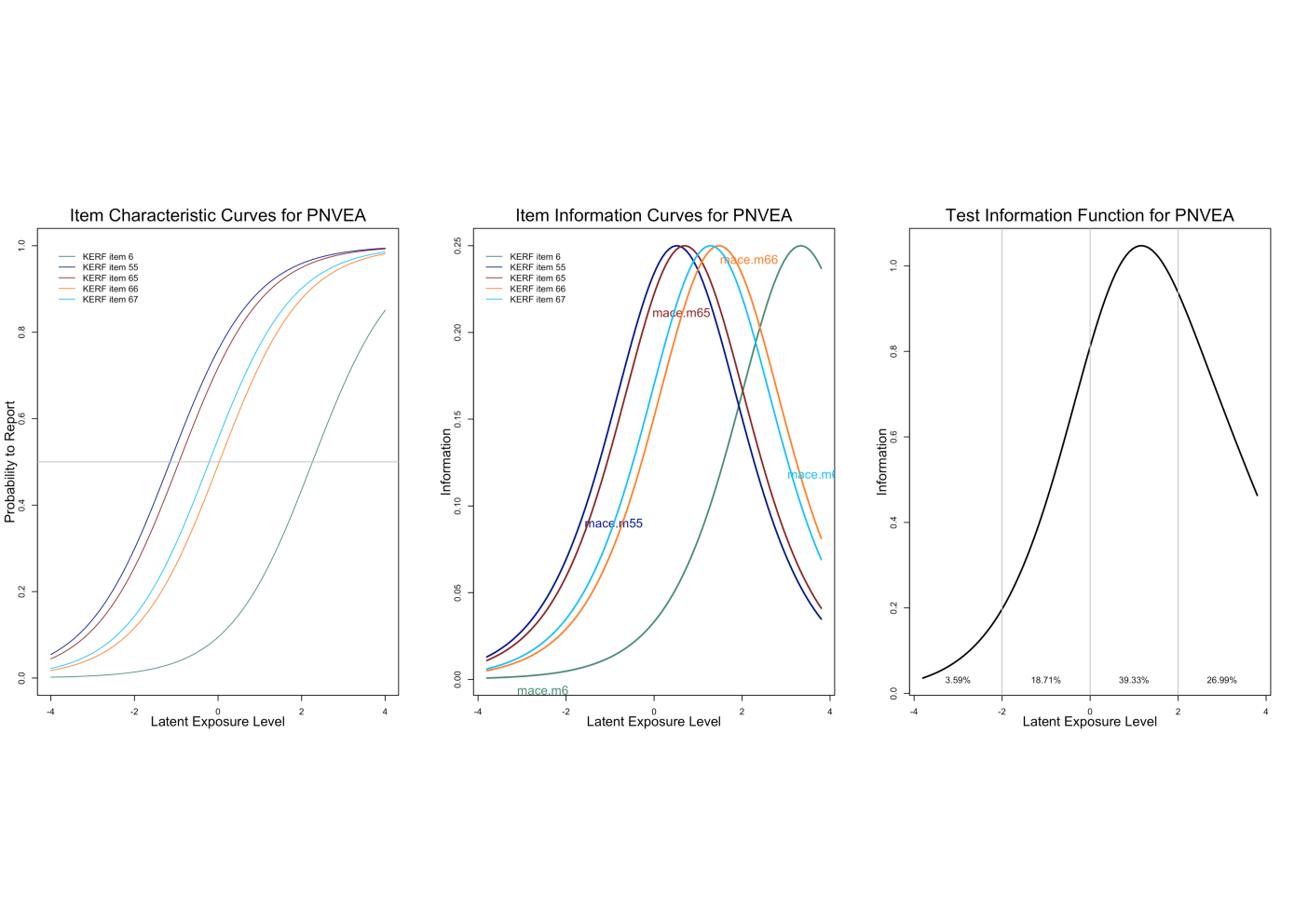
Figure D. Parental-Nonverbal-Emotional Abuse (PNVEA)

Figure E. Parental Physical Abuse (PPA)

**
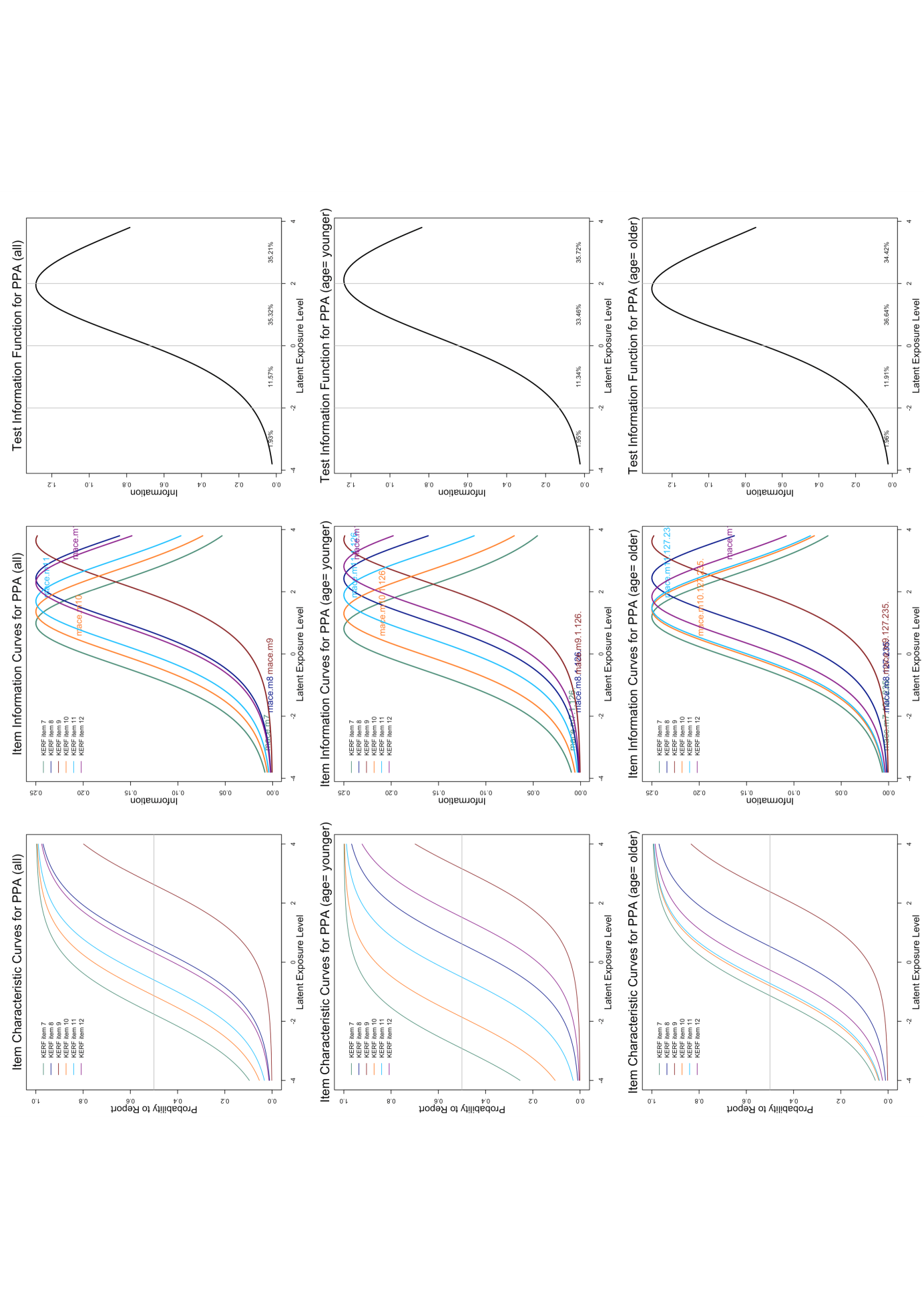
**


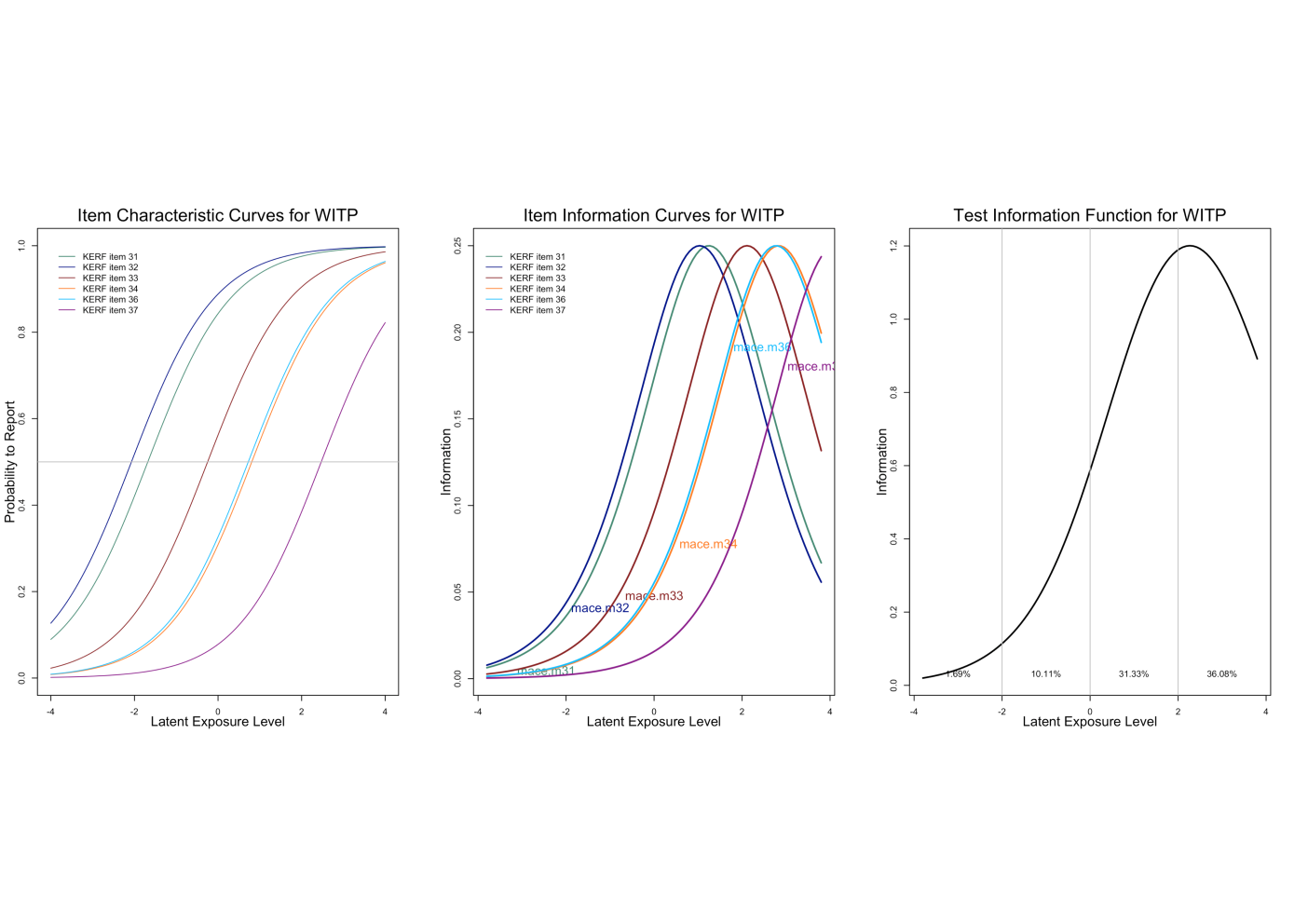
Figure F. Witnessed Violence Towards Parents (WITP)


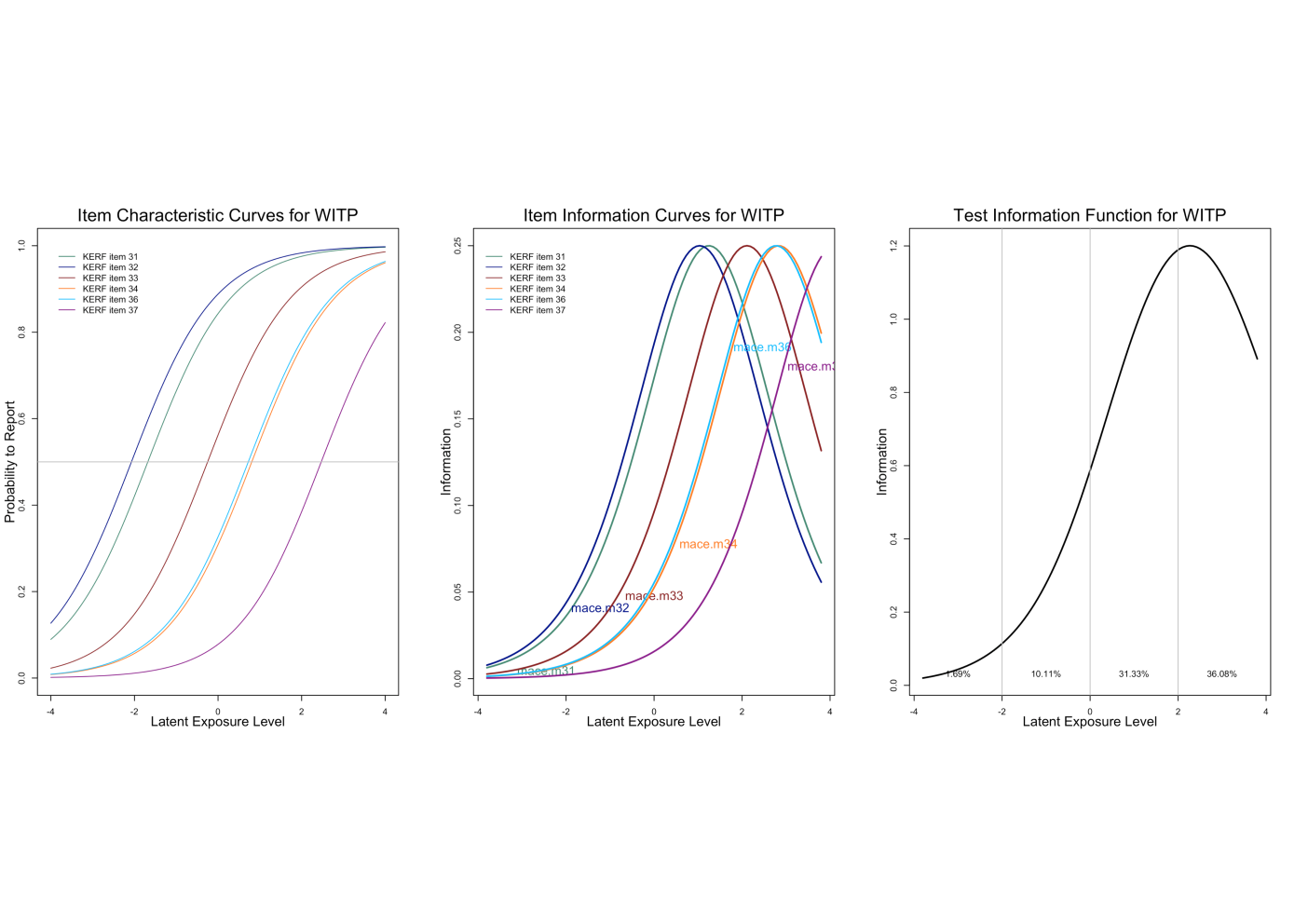
Figure G. Witnessed Violence Towards Siblings (WITS)


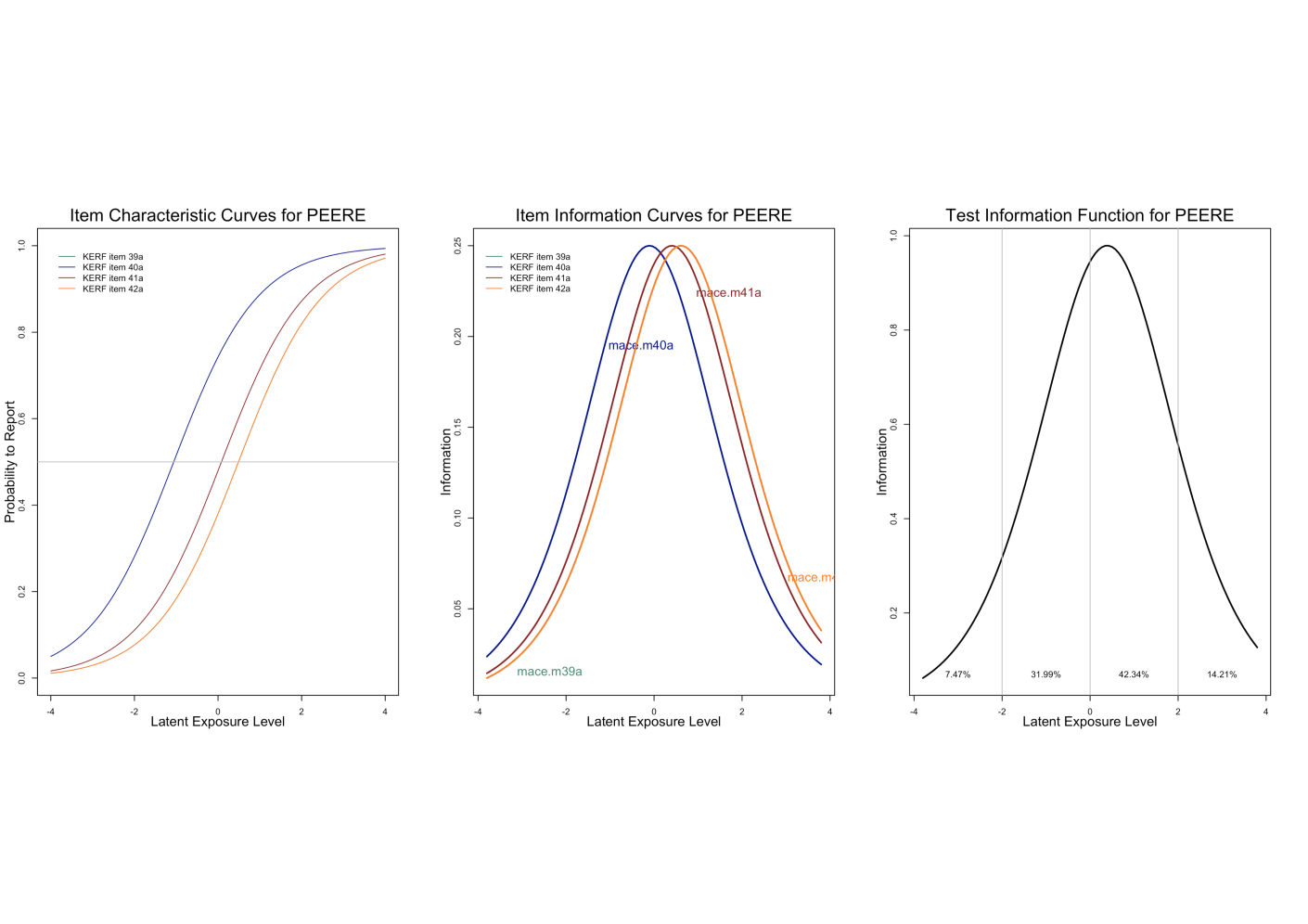
Figure H. Emotional Abuse by Peers (PEERE)


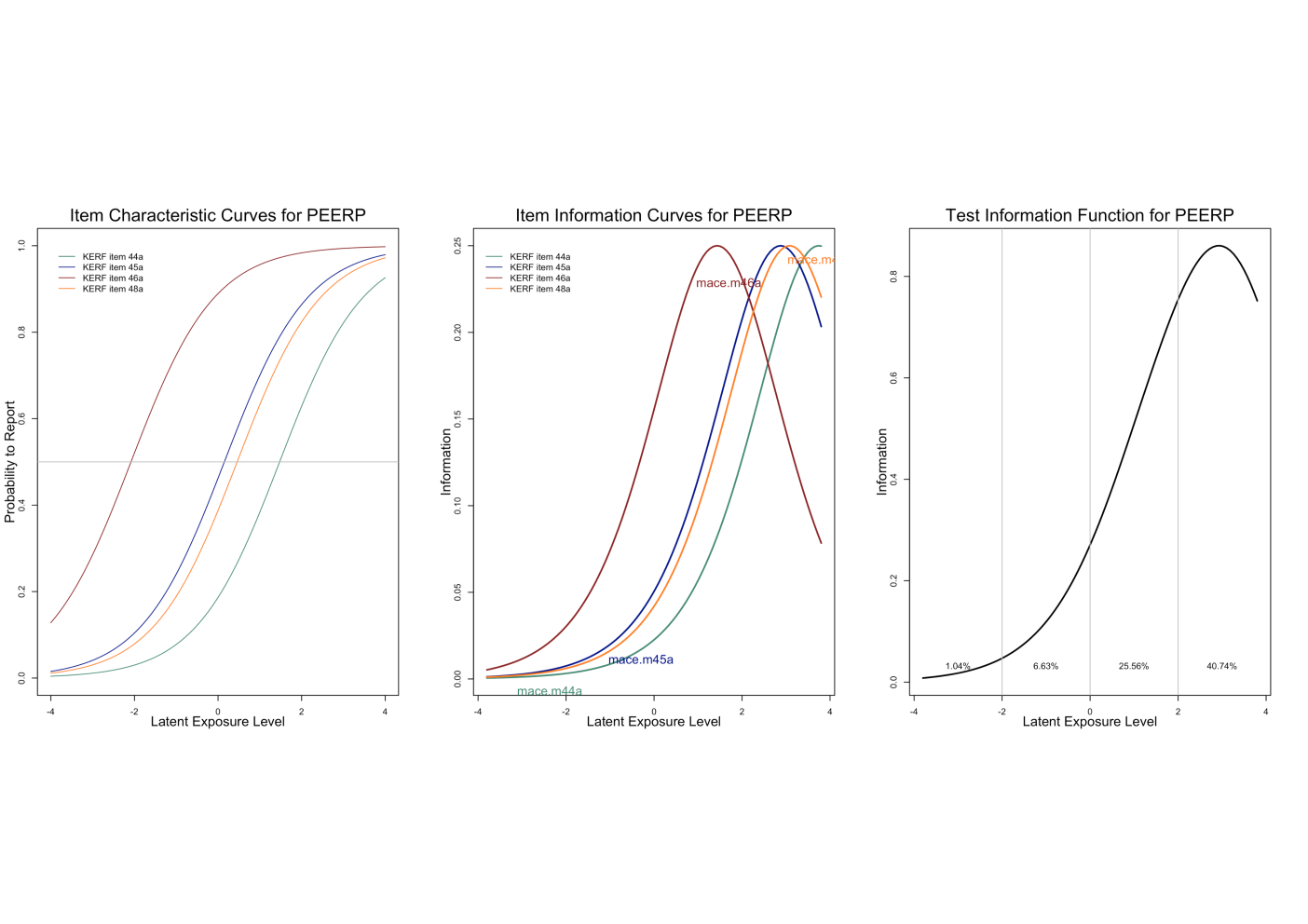
Figure I. Physical Abuse by Peers (PEERP)


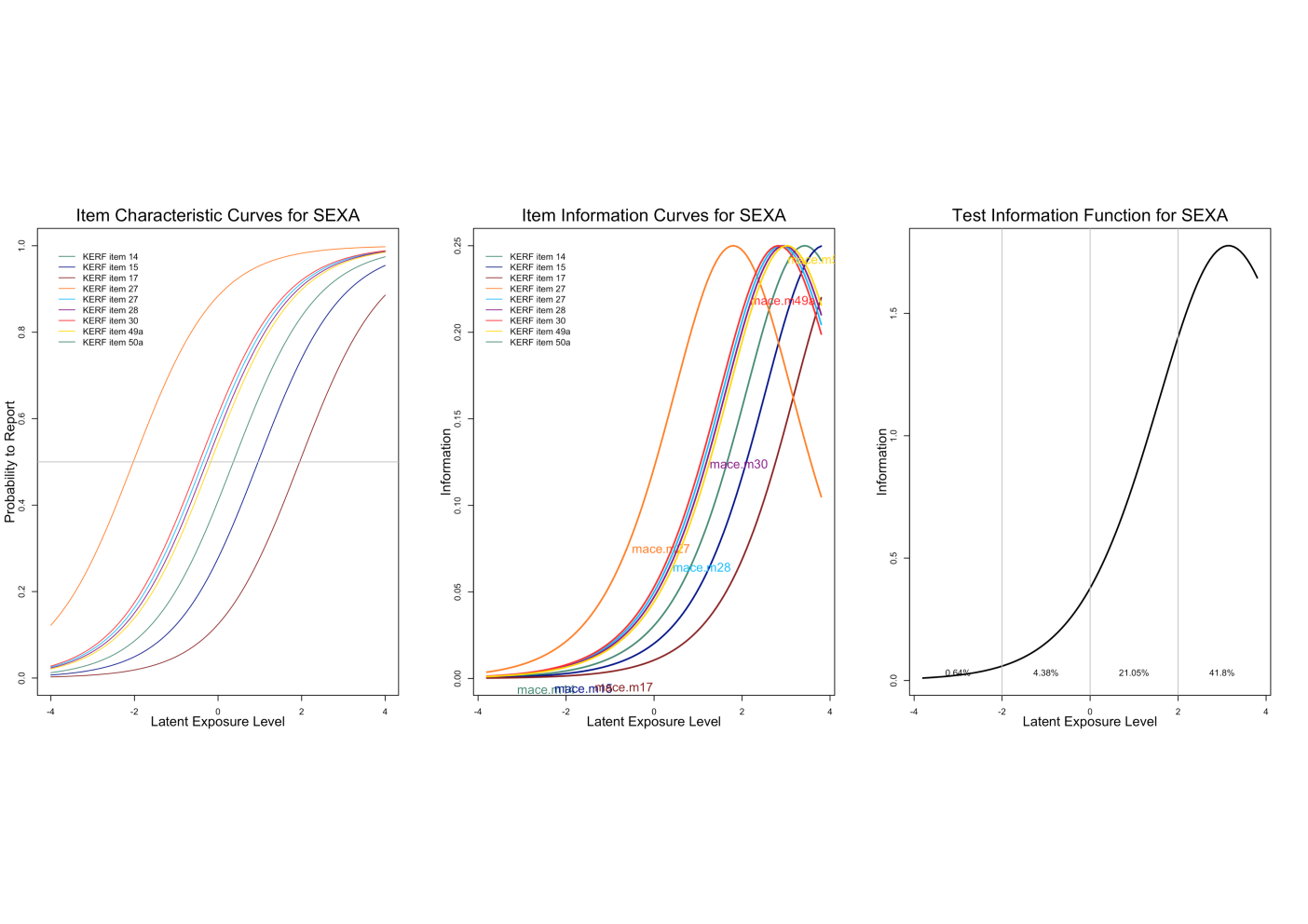
Figure J. Sexual Abuse (SEXA)
