# Supplementary material for: Measuring childhood maltreatment: Psychometric properties of the Norwegian version of the Maltreatment and Abuse Chronology of Exposure (MACE) scale
Source: PLoS One. 2020 Feb 27;15(2):e0229661. doi: 10.1371/journal.pone.0229661 (PMC7046287; doi:10.1371/journal.pone.0229661)
Supplement: S2 File — (DOCX) [file pone.0229661.s002.docx]

**Belastende barndoms- og ungdomserfaringer (MACE)^1^**

**Noen ganger gjør foreldre, steforeldre eller andre voksne som bor sammen vonde ting. Hvis du opplevde dette i din barndom og ungdom (i løpet av de første 18 årene av livet ditt), angi så nøye du kan alderen din da det skjedde. Kryss av for hver aktuell alder!**

***For eksempel****: Ble du skjelt ut av foreldre, steforeldre eller andre som bodde hjemme hos deg? Kalte de deg for ting, og fornærmet deg, som at du var «tjukk», «stygg», «dum» osv. mer enn bare noen få ganger i løpet av året? Hvis din far skjelte deg ut da du var 6-8 år gammel, og din mor fornærmet deg da du var 8-10 år og den nye samboeren til din mor kalte deg for ting da du var 17 år gammel, så krysser du av slik:*

| *1* | *2* | *3* | *4* | *5* | *6* | *7* | *8* | *9* | *10* | *11* | *12* | *13* | *14* | *15* | *16* | *17* | *18* |
| --- | --- | --- | --- | --- | --- | --- | --- | --- | --- | --- | --- | --- | --- | --- | --- | --- | --- |
|  |  |  |  |  | *x* | *x* | *x* | *x* | *x* |  |  |  |  |  |  | *x* |  |

**Spørsmålene 1-14 gjelder for dine foreldre, steforeldre eller andre voksne hjemme hos deg:**

1. Skjelte de deg ut, kalte deg for ting, fornærmet deg som at du var «tjukk», «stygg», «dum»

osv. mer enn bare noen få ganger i løpet av året?

Ja Nei

Kryss av for hver aktuell alder.

| 1 | 2 | 3 | 4 | 5 | 6 | 7 | 8 | 9 | 10 | 11 | 12 | 13 | 14 | 15 | 16 | 17 | 18 |
| --- | --- | --- | --- | --- | --- | --- | --- | --- | --- | --- | --- | --- | --- | --- | --- | --- | --- |
|  |  |  |  |  |  |  |  |  |  |  |  |  |  |  |  |  |  |

1. Sa de sårende ting som gjorde deg trist, skamfull eller ydmyket deg mer enn bare noen

Ja Nei

få ganger i løpet av året?

Kryss av for hver aktuell alder.

| 1 | 2 | 3 | 4 | 5 | 6 | 7 | 8 | 9 | 10 | 11 | 12 | 13 | 14 | 15 | 16 | 17 | 18 |
| --- | --- | --- | --- | --- | --- | --- | --- | --- | --- | --- | --- | --- | --- | --- | --- | --- | --- |
|  |  |  |  |  |  |  |  |  |  |  |  |  |  |  |  |  |  |

1. Skrek de eller brølte de til deg mer enn bare noen få ganger i løpet av året?

Ja Nei

Kryss av for hver aktuell alder.

| 1 | 2 | 3 | 4 | 5 | 6 | 7 | 8 | 9 | 10 | 11 | 12 | 13 | 14 | 15 | 16 | 17 | 18 |
| --- | --- | --- | --- | --- | --- | --- | --- | --- | --- | --- | --- | --- | --- | --- | --- | --- | --- |
|  |  |  |  |  |  |  |  |  |  |  |  |  |  |  |  |  |  |

1. Oppførte de seg på en måte som gjorde deg redd for at du kunne bli fysisk skadet?

Ja Nei

Kryss av for hver aktuell alder.

| 1 | 2 | 3 | 4 | 5 | 6 | 7 | 8 | 9 | 10 | 11 | 12 | 13 | 14 | 15 | 16 | 17 | 18 |
| --- | --- | --- | --- | --- | --- | --- | --- | --- | --- | --- | --- | --- | --- | --- | --- | --- | --- |
|  |  |  |  |  |  |  |  |  |  |  |  |  |  |  |  |  |  |

1. Stengte de deg inne i et skap, på et loft, i en kjeller eller garasje eller på et annet trangt og mørkt sted?

Ja Nei

Kryss av for hver aktuell alder.

| 1 | 2 | 3 | 4 | 5 | 6 | 7 | 8 | 9 | 10 | 11 | 12 | 13 | 14 | 15 | 16 | 17 | 18 |
| --- | --- | --- | --- | --- | --- | --- | --- | --- | --- | --- | --- | --- | --- | --- | --- | --- | --- |
|  |  |  |  |  |  |  |  |  |  |  |  |  |  |  |  |  |  |

1. Knuffet de deg, tok hardt tak i deg, dyttet deg, slo deg med flat hånd i ansiktet eller kløp deg med vilje, slo deg med knyttneven eller sparket deg?

Ja Nei

Kryss av for hver aktuell alder.

| 1 | 2 | 3 | 4 | 5 | 6 | 7 | 8 | 9 | 10 | 11 | 12 | 13 | 14 | 15 | 16 | 17 | 18 |
| --- | --- | --- | --- | --- | --- | --- | --- | --- | --- | --- | --- | --- | --- | --- | --- | --- | --- |
|  |  |  |  |  |  |  |  |  |  |  |  |  |  |  |  |  |  |

1. Slo de deg så hardt at du fikk merker som varte lengre enn noen minutter?

Ja Nei

Kryss av for hver aktuell alder.

| 1 | 2 | 3 | 4 | 5 | 6 | 7 | 8 | 9 | 10 | 11 | 12 | 13 | 14 | 15 | 16 | 17 | 18 |
| --- | --- | --- | --- | --- | --- | --- | --- | --- | --- | --- | --- | --- | --- | --- | --- | --- | --- |
|  |  |  |  |  |  |  |  |  |  |  |  |  |  |  |  |  |  |

1. Slo de deg så hardt eller skadet de deg med vilje på en eller annen måte slik at du trengte legetilsyn eller burde ha fått det?

Ja Nei

Kryss av for hver aktuell alder.

| 1 | 2 | 3 | 4 | 5 | 6 | 7 | 8 | 9 | 10 | 11 | 12 | 13 | 14 | 15 | 16 | 17 | 18 |
| --- | --- | --- | --- | --- | --- | --- | --- | --- | --- | --- | --- | --- | --- | --- | --- | --- | --- |
|  |  |  |  |  |  |  |  |  |  |  |  |  |  |  |  |  |  |

1. Slo de deg med flat hånd på baken, armene eller bena?

Ja Nei

Kryss av for hver aktuell alder.

| 1 | 2 | 3 | 4 | 5 | 6 | 7 | 8 | 9 | 10 | 11 | 12 | 13 | 14 | 15 | 16 | 17 | 18 |
| --- | --- | --- | --- | --- | --- | --- | --- | --- | --- | --- | --- | --- | --- | --- | --- | --- | --- |
|  |  |  |  |  |  |  |  |  |  |  |  |  |  |  |  |  |  |

1. Slo de deg på din nakne bak?

Ja Nei

Kryss av for hver aktuell alder.

| 1 | 2 | 3 | 4 | 5 | 6 | 7 | 8 | 9 | 10 | 11 | 12 | 13 | 14 | 15 | 16 | 17 | 18 |
| --- | --- | --- | --- | --- | --- | --- | --- | --- | --- | --- | --- | --- | --- | --- | --- | --- | --- |
|  |  |  |  |  |  |  |  |  |  |  |  |  |  |  |  |  |  |

1. Slo de deg med en gjenstand, som for eksempel stropp, belte, børste, stokk, rør, kost, sleiv osv.?

Ja Nei

Kryss av for hver aktuell alder.

| 1 | 2 | 3 | 4 | 5 | 6 | 7 | 8 | 9 | 10 | 11 | 12 | 13 | 14 | 15 | 16 | 17 | 18 |
| --- | --- | --- | --- | --- | --- | --- | --- | --- | --- | --- | --- | --- | --- | --- | --- | --- | --- |
|  |  |  |  |  |  |  |  |  |  |  |  |  |  |  |  |  |  |

1. Berørte eller klådde de på kroppen din på en seksuell måte?

Ja Nei

Kryss av for hver aktuell alder.

| 1 | 2 | 3 | 4 | 5 | 6 | 7 | 8 | 9 | 10 | 11 | 12 | 13 | 14 | 15 | 16 | 17 | 18 |
| --- | --- | --- | --- | --- | --- | --- | --- | --- | --- | --- | --- | --- | --- | --- | --- | --- | --- |
|  |  |  |  |  |  |  |  |  |  |  |  |  |  |  |  |  |  |

1. Fikk de deg til å ta på kroppen sin (kroppen til den voksne) på en seksuell måte**?**

Ja Nei

Kryss av for hver aktuell alder.

| 1 | 2 | 3 | 4 | 5 | 6 | 7 | 8 | 9 | 10 | 11 | 12 | 13 | 14 | 15 | 16 | 17 | 18 |
| --- | --- | --- | --- | --- | --- | --- | --- | --- | --- | --- | --- | --- | --- | --- | --- | --- | --- |
|  |  |  |  |  |  |  |  |  |  |  |  |  |  |  |  |  |  |

1. Hadde de på en eller annen måte (oralt, analt eller vaginalt) samleie med deg

(føre penis eller gjenstander inn i skjede, endetarmsåpning eller munn)?

Ja Nei

Kryss av for hver aktuell alder.

| 1 | 2 | 3 | 4 | 5 | 6 | 7 | 8 | 9 | 10 | 11 | 12 | 13 | 14 | 15 | 16 | 17 | 18 |
| --- | --- | --- | --- | --- | --- | --- | --- | --- | --- | --- | --- | --- | --- | --- | --- | --- | --- |
|  |  |  |  |  |  |  |  |  |  |  |  |  |  |  |  |  |  |

______________________________________________________________________________________

**Noen ganger gjør foreldre, steforeldre eller andre voksne som bor sammen vonde ting mot søsken (søster, bror, stesøsken). Hvis du var vitne til dette i din barndom og ungdom (i løpet av de første 18 årene av livet ditt), angi så nøye du kan alderen din da det skjedde. Kryss av for hver aktuell alder!**

1. Ble dine søsken (stesøsken) knuffet, tatt hardt tak i, dyttet, slått med flat hånd

i ansiktet, kløpet med vilje, slått med knyttneven eller sparket?

Ja Nei

**Kryss av for hver aktuell alder.**

| 1 | 2 | 3 | 4 | 5 | 6 | 7 | 8 | 9 | 10 | 11 | 12 | 13 | 14 | 15 | 16 | 17 | 18 |
| --- | --- | --- | --- | --- | --- | --- | --- | --- | --- | --- | --- | --- | --- | --- | --- | --- | --- |
|  |  |  |  |  |  |  |  |  |  |  |  |  |  |  |  |  |  |

1. Ble dine søsken (stesøsken) slått så hardt, at de fikk merker på kroppen som varte lengre enn i noen minutter?

Ja Nei

Kryss av for hver aktuell alder.

| 1 | 2 | 3 | 4 | 5 | 6 | 7 | 8 | 9 | 10 | 11 | 12 | 13 | 14 | 15 | 16 | 17 | 18 |
| --- | --- | --- | --- | --- | --- | --- | --- | --- | --- | --- | --- | --- | --- | --- | --- | --- | --- |
|  |  |  |  |  |  |  |  |  |  |  |  |  |  |  |  |  |  |

1. Ble dine søsken (stesøsken) utsatt for upassende seksuelle kommentarer eller antydninger?

Ja Nei

Kryss av for hver aktuell alder.

| 1 | 2 | 3 | 4 | 5 | 6 | 7 | 8 | 9 | 10 | 11 | 12 | 13 | 14 | 15 | 16 | 17 | 18 |
| --- | --- | --- | --- | --- | --- | --- | --- | --- | --- | --- | --- | --- | --- | --- | --- | --- | --- |
|  |  |  |  |  |  |  |  |  |  |  |  |  |  |  |  |  |  |

1. Ble dine søsken (stesøsken) berørt eller klådd på, på en seksuell måte?

Ja Nei

Kryss av for hver aktuell alder.

| 1 | 2 | 3 | 4 | 5 | 6 | 7 | 8 | 9 | 10 | 11 | 12 | 13 | 14 | 15 | 16 | 17 | 18 |
| --- | --- | --- | --- | --- | --- | --- | --- | --- | --- | --- | --- | --- | --- | --- | --- | --- | --- |
|  |  |  |  |  |  |  |  |  |  |  |  |  |  |  |  |  |  |

1. Ble dine søsken (stesøsken) truet med at de ville bli skadet?

Ja Nei

Kryss av for hver aktuell alder.

| 1 | 2 | 3 | 4 | 5 | 6 | 7 | 8 | 9 | 10 | 11 | 12 | 13 | 14 | 15 | 16 | 17 | 18 |
| --- | --- | --- | --- | --- | --- | --- | --- | --- | --- | --- | --- | --- | --- | --- | --- | --- | --- |
|  |  |  |  |  |  |  |  |  |  |  |  |  |  |  |  |  |  |

______________________________________________________________________________________

**Noen ganger gjør andre voksne eller personer som er eldre enn deg og som IKKE bor hjemme hos deg (altså ikke foreldre, ikke steforeldre, ikke personer som bor hjemme hos deg) vonde ting mot deg. Hvis du opplevde dette i din barndom og ungdom (i løpet av de første 18 årene av livet ditt), angi så nøye du kan alderen din da det skjedde. Kryss av for hver aktuell alder!**

1. Berørte eller klådde de på kroppen din på en seksuell måte?

Ja Nei

Kryss av for hver aktuell alder.

| 1 | 2 | 3 | 4 | 5 | 6 | 7 | 8 | 9 | 10 | 11 | 12 | 13 | 14 | 15 | 16 | 17 | 18 |
| --- | --- | --- | --- | --- | --- | --- | --- | --- | --- | --- | --- | --- | --- | --- | --- | --- | --- |
|  |  |  |  |  |  |  |  |  |  |  |  |  |  |  |  |  |  |

1. Fikk de deg til å ta på kroppen sin (kroppen til den voksne) på en seksuell måte?

Ja Nei

Kryss av for hver aktuell alder.

| 1 | 2 | 3 | 4 | 5 | 6 | 7 | 8 | 9 | 10 | 11 | 12 | 13 | 14 | 15 | 16 | 17 | 18 |
| --- | --- | --- | --- | --- | --- | --- | --- | --- | --- | --- | --- | --- | --- | --- | --- | --- | --- |
|  |  |  |  |  |  |  |  |  |  |  |  |  |  |  |  |  |  |

1. Hadde de på en eller annen måte (oralt, analt eller vaginalt) samleie med deg (føre penis eller gjenstander inn i skjede, endetarmsåpning eller munn)?

Ja Nei

Kryss av for hver aktuell alder.

| 1 | 2 | 3 | 4 | 5 | 6 | 7 | 8 | 9 | 10 | 11 | 12 | 13 | 14 | 15 | 16 | 17 | 18 |
| --- | --- | --- | --- | --- | --- | --- | --- | --- | --- | --- | --- | --- | --- | --- | --- | --- | --- |
|  |  |  |  |  |  |  |  |  |  |  |  |  |  |  |  |  |  |

______________________________________________________________________________________

**Noen ganger blir det heftige krangler eller fysiske konflikter mellom foreldre, steforeldre eller andre voksne som en bor sammen med (for eksempel samboere, besteforeldre).** **Hvis du var vitne til dette i din barndom og ungdom (i løpet av de første 18 årene av livet ditt), angi så nøye du kan alderen din da det skjedde. Kryss av for hver aktuell alder!**

1. Du var vitne til at andre voksne som du bodde sammen med, kranglet heftig med din far (stefar, fosterfar eller bestefar), fornærmet han eller truet med å skade han.

Ja Nei

Kryss av for hver aktuell alder.

| 1 | 2 | 3 | 4 | 5 | 6 | 7 | 8 | 9 | 10 | 11 | 12 | 13 | 14 | 15 | 16 | 17 | 18 |
| --- | --- | --- | --- | --- | --- | --- | --- | --- | --- | --- | --- | --- | --- | --- | --- | --- | --- |
|  |  |  |  |  |  |  |  |  |  |  |  |  |  |  |  |  |  |

1. Du var vitne til at andre voksne som du bodde sammen med, kranglet heftig med din mor (stemor, fostermor eller bestemor), fornærmet henne eller truet med å skade henne.

Ja Nei

Kryss av for hver aktuell alder.

| 1 | 2 | 3 | 4 | 5 | 6 | 7 | 8 | 9 | 10 | 11 | 12 | 13 | 14 | 15 | 16 | 17 | 18 |
| --- | --- | --- | --- | --- | --- | --- | --- | --- | --- | --- | --- | --- | --- | --- | --- | --- | --- |
|  |  |  |  |  |  |  |  |  |  |  |  |  |  |  |  |  |  |

1. Du så at andre voksne som du bodde sammen med, dyttet din mor (stemor, fostermor eller bestemor), tok hardt tak i henne, slo til henne i ansiktet eller kastet ting på henne.

Ja Nei

Kryss av for hver aktuell alder.

| 1 | 2 | 3 | 4 | 5 | 6 | 7 | 8 | 9 | 10 | 11 | 12 | 13 | 14 | 15 | 16 | 17 | 18 |
| --- | --- | --- | --- | --- | --- | --- | --- | --- | --- | --- | --- | --- | --- | --- | --- | --- | --- |
|  |  |  |  |  |  |  |  |  |  |  |  |  |  |  |  |  |  |

1. Du så at andre voksne som du bodde sammen med, slo din mor (stemor, fostermor eller bestemor) så hardt at hun fikk merker på kroppen som varte lengre enn i noen minutter.

Ja Nei

Kryss av for hver aktuell alder.

| 1 | 2 | 3 | 4 | 5 | 6 | 7 | 8 | 9 | 10 | 11 | 12 | 13 | 14 | 15 | 16 | 17 | 18 |
| --- | --- | --- | --- | --- | --- | --- | --- | --- | --- | --- | --- | --- | --- | --- | --- | --- | --- |
|  |  |  |  |  |  |  |  |  |  |  |  |  |  |  |  |  |  |

1. Du så at andre voksne som du bodde sammen med, dyttet din far (stefar, fosterfar eller bestefar), tok hardt tak i ham, slo til han i ansiktet eller kastet ting på han.

Ja Nei

Kryss av for hver aktuell alder.

| 1 | 2 | 3 | 4 | 5 | 6 | 7 | 8 | 9 | 10 | 11 | 12 | 13 | 14 | 15 | 16 | 17 | 18 |
| --- | --- | --- | --- | --- | --- | --- | --- | --- | --- | --- | --- | --- | --- | --- | --- | --- | --- |
|  |  |  |  |  |  |  |  |  |  |  |  |  |  |  |  |  |  |

1. Du så at andre voksne som du bodde sammen med, slo din far (stefar, fosterfar eller bestefar) så hardt at han fikk merker på kroppen som varte lengre enn i noen minutter.

Ja Nei

Kryss av for hver aktuell alder.

| 1 | 2 | 3 | 4 | 5 | 6 | 7 | 8 | 9 | 10 | 11 | 12 | 13 | 14 | 15 | 16 | 17 | 18 |
| --- | --- | --- | --- | --- | --- | --- | --- | --- | --- | --- | --- | --- | --- | --- | --- | --- | --- |
|  |  |  |  |  |  |  |  |  |  |  |  |  |  |  |  |  |  |

______________________________________________________________________________________

**Noen ganger gjør jevnaldrende eller eldre barn/ungdommer vonde ting som for eksempel å mobbe eller plage noen.** **Hvis du opplevde dette i din barndom og ungdom (i løpet av de første 18 årene av livet ditt), angi så nøye du kan alderen din da det skjedde. Kryss av for hver aktuell alder!**

1. De skjelte deg ut, kalte deg for ting, fornærmet deg med at du var «tjukk», «stygg», «dum» osv. mer enn bare få ganger i løpet av året.

Ja Nei

Kryss av for hver aktuell alder.

| 1 | 2 | 3 | 4 | 5 | 6 | 7 | 8 | 9 | 10 | 11 | 12 | 13 | 14 | 15 | 16 | 17 | 18 |
| --- | --- | --- | --- | --- | --- | --- | --- | --- | --- | --- | --- | --- | --- | --- | --- | --- | --- |
|  |  |  |  |  |  |  |  |  |  |  |  |  |  |  |  |  |  |

1. De sa sårende ting som gjorde deg trist, skamfull eller ydmyket deg.

Ja Nei

Kryss av for hver aktuell alder.

| 1 | 2 | 3 | 4 | 5 | 6 | 7 | 8 | 9 | 10 | 11 | 12 | 13 | 14 | 15 | 16 | 17 | 18 |
| --- | --- | --- | --- | --- | --- | --- | --- | --- | --- | --- | --- | --- | --- | --- | --- | --- | --- |
|  |  |  |  |  |  |  |  |  |  |  |  |  |  |  |  |  |  |

1. De baksnakket deg, ydmyket deg offentlig eller spredde rykter om deg.

Ja Nei

Kryss av for hver aktuell alder.

| 1 | 2 | 3 | 4 | 5 | 6 | 7 | 8 | 9 | 10 | 11 | 12 | 13 | 14 | 15 | 16 | 17 | 18 |
| --- | --- | --- | --- | --- | --- | --- | --- | --- | --- | --- | --- | --- | --- | --- | --- | --- | --- |
|  |  |  |  |  |  |  |  |  |  |  |  |  |  |  |  |  |  |

1. De stengte deg ute fra aktiviteter, grupper eller fra felleskapet.

Ja Nei

Kryss av for hver aktuell alder.

| 1 | 2 | 3 | 4 | 5 | 6 | 7 | 8 | 9 | 10 | 11 | 12 | 13 | 14 | 15 | 16 | 17 | 18 |
| --- | --- | --- | --- | --- | --- | --- | --- | --- | --- | --- | --- | --- | --- | --- | --- | --- | --- |
|  |  |  |  |  |  |  |  |  |  |  |  |  |  |  |  |  |  |

1. De truet deg for å få tak i pengene eller eiendelene dine.

Ja Nei

Kryss av for hver aktuell alder.

| 1 | 2 | 3 | 4 | 5 | 6 | 7 | 8 | 9 | 10 | 11 | 12 | 13 | 14 | 15 | 16 | 17 | 18 |
| --- | --- | --- | --- | --- | --- | --- | --- | --- | --- | --- | --- | --- | --- | --- | --- | --- | --- |
|  |  |  |  |  |  |  |  |  |  |  |  |  |  |  |  |  |  |

1. De tvang eller truet deg til å gjøre ting du ikke ville.

Ja Nei

Kryss av for hver aktuell alder.

| 1 | 2 | 3 | 4 | 5 | 6 | 7 | 8 | 9 | 10 | 11 | 12 | 13 | 14 | 15 | 16 | 17 | 18 |
| --- | --- | --- | --- | --- | --- | --- | --- | --- | --- | --- | --- | --- | --- | --- | --- | --- | --- |
|  |  |  |  |  |  |  |  |  |  |  |  |  |  |  |  |  |  |

1. De knuffet deg, tok hardt tak i deg, dyttet deg, slo deg med flat hånd i ansiktet, kløp deg med vilje, slo deg med knyttneven eller sparket deg.

Ja Nei

Kryss av for hver aktuell alder.

| 1 | 2 | 3 | 4 | 5 | 6 | 7 | 8 | 9 | 10 | 11 | 12 | 13 | 14 | 15 | 16 | 17 | 18 |
| --- | --- | --- | --- | --- | --- | --- | --- | --- | --- | --- | --- | --- | --- | --- | --- | --- | --- |
|  |  |  |  |  |  |  |  |  |  |  |  |  |  |  |  |  |  |

1. De slo deg så hardt eller skadet deg på en eller annen måte, slik at du trengte legetilsyn eller burde ha fått det.

Ja Nei

Kryss av for hver aktuell alder.

| 1 | 2 | 3 | 4 | 5 | 6 | 7 | 8 | 9 | 10 | 11 | 12 | 13 | 14 | 15 | 16 | 17 | 18 |
| --- | --- | --- | --- | --- | --- | --- | --- | --- | --- | --- | --- | --- | --- | --- | --- | --- | --- |
|  |  |  |  |  |  |  |  |  |  |  |  |  |  |  |  |  |  |

1. De tvang deg til seksuelle aktiviteter mot din vilje.

Ja Nei

Kryss av for hver aktuell alder.

| 1 | 2 | 3 | 4 | 5 | 6 | 7 | 8 | 9 | 10 | 11 | 12 | 13 | 14 | 15 | 16 | 17 | 18 |
| --- | --- | --- | --- | --- | --- | --- | --- | --- | --- | --- | --- | --- | --- | --- | --- | --- | --- |
|  |  |  |  |  |  |  |  |  |  |  |  |  |  |  |  |  |  |

1. De tvang deg til å utføre bestemte seksuelle handlinger som du ikke ville gjøre.

Ja Nei

Kryss av for hver aktuell alder.

| 1 | 2 | 3 | 4 | 5 | 6 | 7 | 8 | 9 | 10 | 11 | 12 | 13 | 14 | 15 | 16 | 17 | 18 |
| --- | --- | --- | --- | --- | --- | --- | --- | --- | --- | --- | --- | --- | --- | --- | --- | --- | --- |
|  |  |  |  |  |  |  |  |  |  |  |  |  |  |  |  |  |  |

______________________________________________________________________________________

**Oppgi om følgende utsagn passer for deg og din familie opp gjennom barndommen og ungdommen, og** **alderen din da det skjedde. Kryss av for hver aktuell alder!**

1. Du opplevde at selv om din mor (stemor, fostermor eller bestemor) var til stede hjemme, var hun følelsesmessig utilgjengelig for deg av ulike grunner som bruk av rusmidler, alkohol, veldig mye arbeid (arbeidsnarkoman), et kjærlighetsforhold eller at hun hensynsløst fulgte

Ja Nei

egne mål.

Kryss av for hver aktuell alder.

| 1 | 2 | 3 | 4 | 5 | 6 | 7 | 8 | 9 | 10 | 11 | 12 | 13 | 14 | 15 | 16 | 17 | 18 |
| --- | --- | --- | --- | --- | --- | --- | --- | --- | --- | --- | --- | --- | --- | --- | --- | --- | --- |
|  |  |  |  |  |  |  |  |  |  |  |  |  |  |  |  |  |  |

1. Du opplevde at selv om din far (stefar, fosterfar eller bestefar) var til stede hjemme, var han følelsesmessig utilgjengelig for deg av ulike grunner som bruk av rusmidler, alkohol, veldig

Ja Nei

mye arbeid (arbeidsnarkoman), et kjærlighetsforhold eller at han hensynsløst fulgte egne mål.

Kryss av for hver aktuell alder.

| 1 | 2 | 3 | 4 | 5 | 6 | 7 | 8 | 9 | 10 | 11 | 12 | 13 | 14 | 15 | 16 | 17 | 18 |
| --- | --- | --- | --- | --- | --- | --- | --- | --- | --- | --- | --- | --- | --- | --- | --- | --- | --- |
|  |  |  |  |  |  |  |  |  |  |  |  |  |  |  |  |  |  |

1. Det var veldig vanskelig å gjøre en av foreldrene eller en annen viktig foresatt fornøyd.

Ja Nei

Kryss av for hver aktuell alder.

| 1 | 2 | 3 | 4 | 5 | 6 | 7 | 8 | 9 | 10 | 11 | 12 | 13 | 14 | 15 | 16 | 17 | 18 |
| --- | --- | --- | --- | --- | --- | --- | --- | --- | --- | --- | --- | --- | --- | --- | --- | --- | --- |
|  |  |  |  |  |  |  |  |  |  |  |  |  |  |  |  |  |  |

1. En av foreldrene dine eller en annen viktig foresatt hadde ikke tid til eller interesse for å snakke med deg (Hvis dette stemmer, kryss av for Ja).

Ja Nei

Kryss av for hver aktuell alder.

| 1 | 2 | 3 | 4 | 5 | 6 | 7 | 8 | 9 | 10 | 11 | 12 | 13 | 14 | 15 | 16 | 17 | 18 |
| --- | --- | --- | --- | --- | --- | --- | --- | --- | --- | --- | --- | --- | --- | --- | --- | --- | --- |
|  |  |  |  |  |  |  |  |  |  |  |  |  |  |  |  |  |  |

1. Ett eller flere familiemedlemmer fikk deg til å føle deg elsket.

Ja Nei

Kryss av for hver aktuell alder.

| 1 | 2 | 3 | 4 | 5 | 6 | 7 | 8 | 9 | 10 | 11 | 12 | 13 | 14 | 15 | 16 | 17 | 18 |
| --- | --- | --- | --- | --- | --- | --- | --- | --- | --- | --- | --- | --- | --- | --- | --- | --- | --- |
|  |  |  |  |  |  |  |  |  |  |  |  |  |  |  |  |  |  |

1. Ett eller flere familiemedlemmer hjalp deg til å føle deg viktig og spesiell.

Ja Nei

Kryss av for hver aktuell alder.

| 1 | 2 | 3 | 4 | 5 | 6 | 7 | 8 | 9 | 10 | 11 | 12 | 13 | 14 | 15 | 16 | 17 | 18 |
| --- | --- | --- | --- | --- | --- | --- | --- | --- | --- | --- | --- | --- | --- | --- | --- | --- | --- |
|  |  |  |  |  |  |  |  |  |  |  |  |  |  |  |  |  |  |

1. Ett eller flere familiemedlemmer passet på deg og beskyttet deg.

Ja Nei

Kryss av for hver aktuell alder.

| 1 | 2 | 3 | 4 | 5 | 6 | 7 | 8 | 9 | 10 | 11 | 12 | 13 | 14 | 15 | 16 | 17 | 18 |
| --- | --- | --- | --- | --- | --- | --- | --- | --- | --- | --- | --- | --- | --- | --- | --- | --- | --- |
|  |  |  |  |  |  |  |  |  |  |  |  |  |  |  |  |  |  |

1. Ett eller flere familiemedlemmer ville ha fulgt deg til lege eller legevakt når som helst om nødvendig.

Ja Nei

Kryss av for hver aktuell alder.

| 1 | 2 | 3 | 4 | 5 | 6 | 7 | 8 | 9 | 10 | 11 | 12 | 13 | 14 | 15 | 16 | 17 | 18 |
| --- | --- | --- | --- | --- | --- | --- | --- | --- | --- | --- | --- | --- | --- | --- | --- | --- | --- |
|  |  |  |  |  |  |  |  |  |  |  |  |  |  |  |  |  |  |

______________________________________________________________________________________

**Oppgi om følgende utsagn passer for deg og din familie opp gjennom barndommen og ungdommen, og**  **alderen din da det skjedde. Kryss av for hver aktuell alder!**

1. Du hadde ikke nok å spise (Hvis dette stemmer, kryss av for Ja).

Ja Nei

Kryss av for hver aktuell alder.

| 1 | 2 | 3 | 4 | 5 | 6 | 7 | 8 | 9 | 10 | 11 | 12 | 13 | 14 | 15 | 16 | 17 | 18 |
| --- | --- | --- | --- | --- | --- | --- | --- | --- | --- | --- | --- | --- | --- | --- | --- | --- | --- |
|  |  |  |  |  |  |  |  |  |  |  |  |  |  |  |  |  |  |

1. Du måtte gå i uvaskede klær.

Ja Nei

Kryss av for hver aktuell alder.

| 1 | 2 | 3 | 4 | 5 | 6 | 7 | 8 | 9 | 10 | 11 | 12 | 13 | 14 | 15 | 16 | 17 | 18 |
| --- | --- | --- | --- | --- | --- | --- | --- | --- | --- | --- | --- | --- | --- | --- | --- | --- | --- |
|  |  |  |  |  |  |  |  |  |  |  |  |  |  |  |  |  |  |

1. Ingen passet på deg da du var i en alder eller i situasjoner der noen burde ha passet på deg

(Hvis dette stemmer, kryss av for Ja).

Ja Nei

Kryss av for hver aktuell alder.

| 1 | 2 | 3 | 4 | 5 | 6 | 7 | 8 | 9 | 10 | 11 | 12 | 13 | 14 | 15 | 16 | 17 | 18 |
| --- | --- | --- | --- | --- | --- | --- | --- | --- | --- | --- | --- | --- | --- | --- | --- | --- | --- |
|  |  |  |  |  |  |  |  |  |  |  |  |  |  |  |  |  |  |

1. Som barn/ungdom følte du at du måtte ta på deg ansvaret til en voksen.

Ja Nei

Kryss av for hver aktuell alder.

| 1 | 2 | 3 | 4 | 5 | 6 | 7 | 8 | 9 | 10 | 11 | 12 | 13 | 14 | 15 | 16 | 17 | 18 |
| --- | --- | --- | --- | --- | --- | --- | --- | --- | --- | --- | --- | --- | --- | --- | --- | --- | --- |
|  |  |  |  |  |  |  |  |  |  |  |  |  |  |  |  |  |  |

1. Som barn/ungdom følte du at din familie hadde store økonomiske problemer (for eksempel for lite penger, gjeld, fattigdom).

Ja Nei

Kryss av for hver aktuell alder.

| 1 | 2 | 3 | 4 | 5 | 6 | 7 | 8 | 9 | 10 | 11 | 12 | 13 | 14 | 15 | 16 | 17 | 18 |
| --- | --- | --- | --- | --- | --- | --- | --- | --- | --- | --- | --- | --- | --- | --- | --- | --- | --- |
|  |  |  |  |  |  |  |  |  |  |  |  |  |  |  |  |  |  |

1. En eller flere familiemedlemmer holdt viktige ting eller fakta hemmelig for deg.

Ja Nei

Kryss av for hver aktuell alder.

| 1 | 2 | 3 | 4 | 5 | 6 | 7 | 8 | 9 | 10 | 11 | 12 | 13 | 14 | 15 | 16 | 17 | 18 |
| --- | --- | --- | --- | --- | --- | --- | --- | --- | --- | --- | --- | --- | --- | --- | --- | --- | --- |
|  |  |  |  |  |  |  |  |  |  |  |  |  |  |  |  |  |  |

1. Medlemmene i familien din tok vare på hverandre.

Ja Nei

Kryss av for hver aktuell alder.

| 1 | 2 | 3 | 4 | 5 | 6 | 7 | 8 | 9 | 10 | 11 | 12 | 13 | 14 | 15 | 16 | 17 | 18 |
| --- | --- | --- | --- | --- | --- | --- | --- | --- | --- | --- | --- | --- | --- | --- | --- | --- | --- |
|  |  |  |  |  |  |  |  |  |  |  |  |  |  |  |  |  |  |

1. Medlemmene i familien din følte nærhet til hverandre.

Ja Nei

Kryss av for hver aktuell alder.

| 1 | 2 | 3 | 4 | 5 | 6 | 7 | 8 | 9 | 10 | 11 | 12 | 13 | 14 | 15 | 16 | 17 | 18 |
| --- | --- | --- | --- | --- | --- | --- | --- | --- | --- | --- | --- | --- | --- | --- | --- | --- | --- |
|  |  |  |  |  |  |  |  |  |  |  |  |  |  |  |  |  |  |

1. Din familie var en kilde til styrke og støtte for deg.

Ja Nei

Kryss av for hver aktuell alder.

| 1 | 2 | 3 | 4 | 5 | 6 | 7 | 8 | 9 | 10 | 11 | 12 | 13 | 14 | 15 | 16 | 17 | 18 |
| --- | --- | --- | --- | --- | --- | --- | --- | --- | --- | --- | --- | --- | --- | --- | --- | --- | --- |
|  |  |  |  |  |  |  |  |  |  |  |  |  |  |  |  |  |  |

*********************************************************************************
