# Supplementary material for: Measuring childhood maltreatment: Psychometric properties of the Norwegian version of the Maltreatment and Abuse Chronology of Exposure (MACE) scale
Source: PLoS One. 2020 Feb 27;15(2):e0229661. doi: 10.1371/journal.pone.0229661 (PMC7046287; doi:10.1371/journal.pone.0229661)
Supplement: S3 File — (DOCX) [file pone.0229661.s003.docx]

***Innledende spørsmål til skjemaet «Belastende barndoms- og ungdomserfaringer (MACE^1^)»***

Først ønsker vi å stille deg noen spørsmål om **deg, din familie** og de **menneskene som du bodde sammen med i din barndom og ungdom.**

**E1: Hvor gammel er du?** _____________år **Kjønn** Mann Kvinne

| **E2: Hvilke personer** hørte til **familien din** i din barndom og ungdom (i løpet av de første  18 årene av livet ditt)? | E3: **Hvem** var **din(e) hovedomsorgsperson(er)** i din barndom og ungdom (i løpet av de første 18 årene av livet ditt)? |
| --- | --- |
| Mor | Mor |
| Stemor | Stemor |
| Far | Far |
| Stefar | Stefar |
| Søsken | Søsken |
| Stesøsken | Stesøsken |
| Bestemor | Bestemor |
| Bestefar | Bestefar |
| Fosterforeldre/Adoptivforeldre | Fosterforeldre/Adoptivforeldre |
| Fostersøsken/Adoptivsøsken | Fostersøsken/Adoptivsøsken |
| Øvrige slektninger:____________________ | Øvrige slektninger:____________________ |
| Øvrige personer (for eks. omsorgspersoner i institusjon):____________________________ | Øvrige personer (for eks. omsorgspersoner i institusjon eller naboer):_________________ |

^1^Engelsk «Maltreatment and abuse chronology of exposure scale» (MACE), ved Martin H. Teicher, McLean Hospital /Harvard Medical School, USA; Tysk MACE ved Thekkumthala D., Parigger A., Ruf M., Elbert T. & Schauer M, Universität Konstanz, Tyskland; Norsk versjon (*kontaktpersoner): Fosse R.^1*^ ([roar.fosse@vestreviken.no](mailto:roar.fosse@vestreviken.no)), Skjelstad D. V.^1,2*^([dag.skjelstad@vestreviken.no](mailto:dag.skjelstad@vestreviken.no)), Schalinski I.^3^**,** Thekkumthala D.^3^, Elbert T.^3^, Aanondsen C. M.^4,5^, Klæboe Greger H.^4,5*^ ([hanne.k.greger@ntnu.no](mailto:hanne.k.greger@ntnu.no)), Jozefiak T.^4^; ^1^Vestre Viken helseforetak. ^2^Universitetet i Oslo. ^3^Universitetet i Konstanz, Tyskland. ^4^NTNU, ^5^St. Olavs Universitetssykehus.

**E4: Hvem bodde du sammen med** i din barndom og ungdom (i løpet av de første 18 årene av livet ditt?) **Bare nevn personer som du bodde sammen med i minst noen år.**  Kryss av for hver aktuell alder.

| Mor   \| 1 \| 2 \| 3 \| 4 \| 5 \| 6 \| 7 \| 8 \| 9 \| 10 \| 11 \| 12 \| 13 \| 14 \| 15 \| 16 \| 17 \| 18 \| \| --- \| --- \| --- \| --- \| --- \| --- \| --- \| --- \| --- \| --- \| --- \| --- \| --- \| --- \| --- \| --- \| --- \| --- \| \|  \|  \|  \|  \|  \|  \|  \|  \|  \|  \|  \|  \|  \|  \|  \|  \|  \|  \| | Stesøsken (barna til fars/mors nye samboer)   \| 1 \| 2 \| 3 \| 4 \| 5 \| 6 \| 7 \| 8 \| 9 \| 10 \| 11 \| 12 \| 13 \| 14 \| 15 \| 16 \| 17 \| 18 \| \| --- \| --- \| --- \| --- \| --- \| --- \| --- \| --- \| --- \| --- \| --- \| --- \| --- \| --- \| --- \| --- \| --- \| --- \| \|  \|  \|  \|  \|  \|  \|  \|  \|  \|  \|  \|  \|  \|  \|  \|  \|  \|  \| |
| --- | --- | --- | --- | --- | --- | --- | --- | --- | --- | --- | --- | --- | --- | --- | --- | --- | --- | --- | --- | --- | --- | --- | --- | --- | --- | --- | --- | --- | --- | --- | --- | --- | --- | --- | --- | --- | --- | --- | --- | --- | --- | --- | --- | --- | --- | --- | --- | --- | --- | --- | --- | --- | --- | --- | --- | --- | --- | --- | --- | --- | --- | --- | --- | --- | --- | --- | --- | --- | --- | --- | --- | --- | --- |
| Far   \| 1 \| 2 \| 3 \| 4 \| 5 \| 6 \| 7 \| 8 \| 9 \| 10 \| 11 \| 12 \| 13 \| 14 \| 15 \| 16 \| 17 \| 18 \| \| --- \| --- \| --- \| --- \| --- \| --- \| --- \| --- \| --- \| --- \| --- \| --- \| --- \| --- \| --- \| --- \| --- \| --- \| \|  \|  \|  \|  \|  \|  \|  \|  \|  \|  \|  \|  \|  \|  \|  \|  \|  \|  \| | Fostersøsken/Adoptivsøsken   \| 1 \| 2 \| 3 \| 4 \| 5 \| 6 \| 7 \| 8 \| 9 \| 10 \| 11 \| 12 \| 13 \| 14 \| 15 \| 16 \| 17 \| 18 \| \| --- \| --- \| --- \| --- \| --- \| --- \| --- \| --- \| --- \| --- \| --- \| --- \| --- \| --- \| --- \| --- \| --- \| --- \| \|  \|  \|  \|  \|  \|  \|  \|  \|  \|  \|  \|  \|  \|  \|  \|  \|  \|  \| |
| Søsken   \| 1 \| 2 \| 3 \| 4 \| 5 \| 6 \| 7 \| 8 \| 9 \| 10 \| 11 \| 12 \| 13 \| 14 \| 15 \| 16 \| 17 \| 18 \| \| --- \| --- \| --- \| --- \| --- \| --- \| --- \| --- \| --- \| --- \| --- \| --- \| --- \| --- \| --- \| --- \| --- \| --- \| \|  \|  \|  \|  \|  \|  \|  \|  \|  \|  \|  \|  \|  \|  \|  \|  \|  \|  \| | Bestemor   \| 1 \| 2 \| 3 \| 4 \| 5 \| 6 \| 7 \| 8 \| 9 \| 10 \| 11 \| 12 \| 13 \| 14 \| 15 \| 16 \| 17 \| 18 \| \| --- \| --- \| --- \| --- \| --- \| --- \| --- \| --- \| --- \| --- \| --- \| --- \| --- \| --- \| --- \| --- \| --- \| --- \| \|  \|  \|  \|  \|  \|  \|  \|  \|  \|  \|  \|  \|  \|  \|  \|  \|  \|  \| |
| Mors nye samboer   \| 1 \| 2 \| 3 \| 4 \| 5 \| 6 \| 7 \| 8 \| 9 \| 10 \| 11 \| 12 \| 13 \| 14 \| 15 \| 16 \| 17 \| 18 \| \| --- \| --- \| --- \| --- \| --- \| --- \| --- \| --- \| --- \| --- \| --- \| --- \| --- \| --- \| --- \| --- \| --- \| --- \| \|  \|  \|  \|  \|  \|  \|  \|  \|  \|  \|  \|  \|  \|  \|  \|  \|  \|  \| | Bestefar   \| 1 \| 2 \| 3 \| 4 \| 5 \| 6 \| 7 \| 8 \| 9 \| 10 \| 11 \| 12 \| 13 \| 14 \| 15 \| 16 \| 17 \| 18 \| \| --- \| --- \| --- \| --- \| --- \| --- \| --- \| --- \| --- \| --- \| --- \| --- \| --- \| --- \| --- \| --- \| --- \| --- \| \|  \|  \|  \|  \|  \|  \|  \|  \|  \|  \|  \|  \|  \|  \|  \|  \|  \|  \| |
| Mors skiftende samboere   \| 1 \| 2 \| 3 \| 4 \| 5 \| 6 \| 7 \| 8 \| 9 \| 10 \| 11 \| 12 \| 13 \| 14 \| 15 \| 16 \| 17 \| 18 \| \| --- \| --- \| --- \| --- \| --- \| --- \| --- \| --- \| --- \| --- \| --- \| --- \| --- \| --- \| --- \| --- \| --- \| --- \| \|  \|  \|  \|  \|  \|  \|  \|  \|  \|  \|  \|  \|  \|  \|  \|  \|  \|  \| | Øvrige slektninger: ____________________   \| 1 \| 2 \| 3 \| 4 \| 5 \| 6 \| 7 \| 8 \| 9 \|  \| 10 \| 11 \| 12 \| 13 \| 14 \| 15 \| 16 \| 17 \| 18 \| \| --- \| --- \| --- \| --- \| --- \| --- \| --- \| --- \| --- \| --- \| --- \| --- \| --- \| --- \| --- \| --- \| --- \| --- \| --- \| \|  \|  \|  \|  \|  \|  \|  \|  \|  \|  \|  \|  \|  \|  \|  \|  \|  \|  \|  \| |
| Fars nye samboer   \| 1 \| 2 \| 3 \| 4 \| 5 \| 6 \| 7 \| 8 \| 9 \| 10 \| 11 \| 12 \| 13 \| 14 \| 15 \| 16 \| 17 \| 18 \| \| --- \| --- \| --- \| --- \| --- \| --- \| --- \| --- \| --- \| --- \| --- \| --- \| --- \| --- \| --- \| --- \| --- \| --- \| \|  \|  \|  \|  \|  \|  \|  \|  \|  \|  \|  \|  \|  \|  \|  \|  \|  \|  \| | Fosterforeldre/Adoptivforeldre   \| 1 \| 2 \| 3 \| 4 \| 5 \| 6 \| 7 \| 8 \| 9 \| 10 \| 11 \| 12 \| 13 \| 14 \| 15 \| 16 \| 17 \| 18 \| \| --- \| --- \| --- \| --- \| --- \| --- \| --- \| --- \| --- \| --- \| --- \| --- \| --- \| --- \| --- \| --- \| --- \| --- \| \|  \|  \|  \|  \|  \|  \|  \|  \|  \|  \|  \|  \|  \|  \|  \|  \|  \|  \| |
| Fars skiftende samboere   \| 1 \| 2 \| 3 \| 4 \| 5 \| 6 \| 7 \| 8 \| 9 \| 10 \| 11 \| 12 \| 13 \| 14 \| 15 \| 16 \| 17 \| 18 \| \| --- \| --- \| --- \| --- \| --- \| --- \| --- \| --- \| --- \| --- \| --- \| --- \| --- \| --- \| --- \| --- \| --- \| --- \| \|  \|  \|  \|  \|  \|  \|  \|  \|  \|  \|  \|  \|  \|  \|  \|  \|  \|  \| | Øvrige personer (f.eks. omsorgspersoner i institusjon):_____________________________   \| 1 \| 2 \| 3 \| 4 \| 5 \| 6 \| 7 \| 8 \| 9 \| 10 \| 11 \| 12 \| 13 \| 14 \| 15 \| 16 \| 17 \| 18 \| \| --- \| --- \| --- \| --- \| --- \| --- \| --- \| --- \| --- \| --- \| --- \| --- \| --- \| --- \| --- \| --- \| --- \| --- \| \|  \|  \|  \|  \|  \|  \|  \|  \|  \|  \|  \|  \|  \|  \|  \|  \|  \|  \| |

**E5: Det var ingen andre barn som bodde sammen med deg** (ingen søsken, ste-/fostersøsken, etc.)

Stemmer Stemmer ikke

**E6: Hadde du en kjæreste før du fylte 18 år?**

Ja Nei

Kryss av for hver aktuell alder.

| 1 | 2 | 3 | 4 | 5 | 6 | 7 | 8 | 9 | 10 | 11 | 12 | 13 | 14 | 15 | 16 | 17 | 18 |
| --- | --- | --- | --- | --- | --- | --- | --- | --- | --- | --- | --- | --- | --- | --- | --- | --- | --- |
|  |  |  |  |  |  |  |  |  |  |  |  |  |  |  |  |  |  |
