# Supplementary material for: Measuring childhood maltreatment: Psychometric properties of the Norwegian version of the Maltreatment and Abuse Chronology of Exposure (MACE) scale
Source: PLoS One. 2020 Feb 27;15(2):e0229661. doi: 10.1371/journal.pone.0229661 (PMC7046287; doi:10.1371/journal.pone.0229661)
Supplement: Attachment — Submitted filename: Response to reviewers.docx [file pone.0229661.s007.docx]

**Response to reviewers**

PONE-D-19-31983
Fosse et al: Measuring childhood maltreatment: psychometric properties of the Norwegian version of the Maltreatment and Abuse Chronology of Exposure (MACE) scale.

Dear Editor,

Below, please find our response to the comments from the reviewers.

Sincerely yours

Roar Fosse

**Reviewer 1**

R1.1 “Although it comes across as such but its not clearly stated that the scale was developed as a self-report measure.”

Author response: We have added specifications that we administered the instrument as a self-report measure. See p. 2 (line 30), p. 5 (line 100), p. 31 (line 516).

**Reviewer 2**

R2.1 “…since the authors reduced the original scale from 75 to 55 items, they built a new version of the instrument. Therefore I strongly suggest to called this new instrument brief MACE, or modified MACE or something that could identify that the authors are working with a modified instrument (including the title).”

Author response: We have discussed this issue thoroughly within our group, and we find it best to continue with the wording/ labeling for MACE instruments that were used by the original developers and in subsequent US/ German publications. Here, the complete, initial, 75-item experimental version of the Maltreatment and Abuse Chronology of Exposure Scale is denoted as “MACE-X”, and subsequent versions, extracted from the MACE-X, have simply been labeled “MACE”. Our 55-item version lies very close to the “main” American 52-item version of MACE, and by being slightly more extensive than this version, we cannot justifiably label it as “brief”. On this basis, to denote the Norwegian version of MACE that we have developed, we now consistently write “Norwegian MACE” or “Norwegian MACE-55”, and some places only “MACE-55” or “MACE” if the context makes it clear that we talk about the Norwegian version. Furthermore, we denote the original, complete, experimental 75-item version as “MACE-X”. In addition, we have made changes to the manuscript at places where we ourselves had used the term “brief” to denote the Norwegian MACE. See R2.1. on: p. 2 (lines 27-30), p. 4 (lines 77-78 and 88-94), p. 5 (lines 100-105, 116), p. 6 (line 122, 129), p. 7 (line 159), p. 10 (lines 229, 233), p. 11 (line 255), p. 22 (lines 381-382), p. 24 (line 396).

**Other changes**

We have changed the affiliation of our third author on the paper, Inga Schalinski, to: “Charité – Universitätsmedizin Berlin, corporate member of Freie Universität Berlin, Humboldt-Universität zu Berlin, and Berlin Institute of Health (BIH), Institute of Medical Psychology, Berlin, Germany”
